# Effectiveness and Feasibility of Internet-Based Interventions for Grief After Bereavement: Systematic Review and Meta-analysis

**DOI:** 10.2196/29661

**Published:** 2021-12-08

**Authors:** Andrea E Zuelke, Melanie Luppa, Margrit Löbner, Alexander Pabst, Christine Schlapke, Janine Stein, Steffi G Riedel-Heller

**Affiliations:** 1 Institute of Social Medicine, Occupational Health and Public Health (ISAP) Medical Faculty, University of Leipzig Leipzig Germany

**Keywords:** grief, systematic review, meta-analysis, internet-based, online therapy

## Abstract

**Background:**

Although grief and its symptoms constitute a normal reaction to experiences of loss, some of those affected still report elevated levels of distress after an extended period, often termed complicated grief. Beneficial treatment effects of face-to-face therapies, for example, grief counseling or cognitive behavioral therapy against complicated grief, have been reported. Evaluations of internet- and mobile-based interventions targeting symptoms of grief in bereaved individuals with regard to objective quality criteria are currently lacking.

**Objective:**

We aim to conduct a systematic review and meta-analysis on the effectiveness and feasibility of internet- and mobile-based interventions against symptoms of grief after bereavement.

**Methods:**

We conducted systematic literature searches of randomized controlled trials or feasibility studies published before January 9, 2020, following PRISMA (Preferred Reporting Items for Systematic Reviews and Meta-Analyses) guidelines, in PubMed, PsycINFO, Web of Science Core Collection, and the Cochrane Library. The quality of evidence was assessed using the Grading of Recommendations, Assessment, Development, and Evaluations system. We further assessed aspects of feasibility and rated quality of interventions using criteria suggested by an expert panel on mental health care (German Association for Psychiatry, Psychotherapy, and Psychosomatics). A random-effects meta-analysis was conducted to assess between-group effect sizes.

**Results:**

In total, 9 trials (N=1349) were included. Of these, 7 studies were analyzed meta-analytically. Significant effects were found for symptoms of grief (*g*=0.54, 95% CI 0.32-0.77), depression (*g*=0.44, 95% CI 0.20-0.68), and posttraumatic stress (*g*=0.82, 95% CI 0.63-1.01). Heterogeneity was moderate for grief and depression (*I*^2^=48.75% and 55.19%, respectively) and low for posttraumatic stress symptoms (*I*^2^=0%). The overall quality of evidence was graded low (grief and depression) to moderate (posttraumatic stress). User satisfaction with the interventions was high, as was the quality of the interventions assessed using objective quality criteria.

**Conclusions:**

Internet- or mobile-based interventions might constitute an effective treatment approach against symptoms of grief in bereaved adults. However, the small sample sizes and limited number of studies included in the review warrant further investigation.

**Trial Registration:**

International Prospective Register of Systematic Reviews (PROSPERO) CRD42012002100; https://www.crd.york.ac.uk/prospero/display_record.php?RecordID=131428

## Introduction

### Background

Owing to the increasing use of the internet, internet- and mobile-based interventions (IMIs) offer valuable treatment options for a broad range of mental health diagnoses and syndromes available to sections of the society [1]. The effectiveness of IMIs has already been proven for mild to moderate depression [2-4], anxiety [5,6], posttraumatic stress disorder [7-9], and other mental health diagnoses [10]. Reviews have reported effect sizes comparable with those observed in face-to-face therapies [11]. Compared with face-to-face contact and traditional therapies, IMIs offer several advantages, including low-threshold accessibility, flexible use independent of time and location, and high levels of anonymity and privacy, which might be especially useful for people with fear of stigmatization as a result of mental illness [12-15]. For these and other reasons, IMIs provide a feasible approach to reach underserved populations, such as older citizens or people living in rural areas with possibly difficult access to mental health care services.

Grief and its symptoms have long been recognized as a normal reaction to the loss of a significant other [16,17]. Although most bereaved individuals are eventually able to accept the loss and cope with their grief after a certain amount of time, some still report elevated levels of distress, such as posttraumatic stress, depressive symptoms, and persistent symptoms of grief after an extended period (ie, ≥6 months after the loss or longer) [18,19]. It is estimated that these persisting symptoms of loss-related grief, often termed complicated or prolonged grief, are present in 6%-10% of those experiencing bereavement [20]. Previous reviews and meta-analyses have reported beneficial treatment effects of face-to-face interventions, for example, grief counseling or cognitive behavioral therapy (CBT) against complicated grief [19,21,22]. However, a treatment gap for bereaved individuals has been suspected [23-25], further stressing the potential of IMIs as a safe and effective treatment option.

### Objectives

So far, interventions targeting symptoms of grief in bereaved individuals have not been evaluated with regard to objective quality criteria. Assessing the quality of IMIs targeting symptoms of grief after bereavement could therefore help establish IMIs as a feasible treatment option in the health care sector.

Against this background, this review aims to do the following:

1. Provide evidence on the effectiveness of IMIs in targeting symptoms of grief after bereavement. The rationale for the review and meta-analysis was determined in advance in a published review protocol [26].
2. Critically assess the quality of available evidence using a well-established standardized tool for methodological quality assessment, the Grading of Recommendations, Assessment, Development, and Evaluations (GRADE) system [27].
3. Assess the quality of applied interventions using objective quality criteria proposed by an expert panel on mental health care, namely, the Deutsche Gesellschaft für Psychiatrie und Psychotherapie, Psychosomatik und Nervenheilkunde (DGPPN; German Association for Psychiatry, Psychotherapy, and Pschosomatics) [1], thereby allowing for statements on clinical implications and the potential of IMIs for individuals experiencing grief after bereavement.
4. Provide information on feasibility of treatment and satisfaction of trial participants. This will provide valuable information on the potential of IMIs for both clinicians and decision makers in mental health care as well as for individuals experiencing grief after bereavement.

## Methods

### Registration, Protocol, and Guidelines

The review methods, eligibility criteria, and strategy for data analyses are outlined in the study protocol [26]. The systematic review was registered with PROSPERO (CRD42012002100). We followed the recommendations of PRISMA (Preferred Reporting items for Systematic Reviews and Meta-Analyses) guidelines [28].

### Eligibility Criteria

We searched for randomized controlled trials (RCTs) and feasibility studies published before January 9, 2020, including adults (≥18 years) in bereavement. Measures of the effectiveness and feasibility of IMIs were included. IMIs were defined as any psychological intervention targeting bereavement provided in a web-based or mobile setting, defined as online-, internet-, web-, or mobile-based. Studies were excluded if the intervention was an online self-help support group, forum, or chat or an internet- or mobile-based lifestyle intervention, that is, interventions aimed at increasing quality of life or overall well-being but not targeting symptoms of specific mental health conditions. The respective IMIs had to be compared with another IMI or to one of the following control conditions: no psychological treatment, attention or psychological placebo, waiting list, and active or no IMI treatment.

To be eligible for the review, original studies had to be targeted at individuals who experienced bereavement, whereas grief or grief-related symptoms were required as outcomes.

### Search Strategy and Study Selection

A database search was conducted using a comprehensive search strategy for MEDLINE (PubMed interface), Cochrane Central Register of Controlled Trials, PsycINFO, and Web of Science (Web of Science interface). Studies published in English or German were considered. A combination of the following search terms was used*: bereavement or widowhood or grief* AND *online or web or computer or mobile or e-health or internet* AND *intervention or psychotherapy or cognitive behavioral therapy or cbt.* If feasible, Medical Subject Headings were used as search terms. The finalized MEDLINE search strategy was adapted to the syntax and subject heading specifications of the other databases. The search details for MEDLINE are available in Multimedia Appendix 1.

First, titles and abstracts were screened for all database returns by 2 researchers independently (M Luppa and CS). Second, studies were checked according to the following eligibility criteria by full-text analysis: (1) published in English or German, (2) participants aged ≥18 years, (3) participants experienced bereavement, (4) an IMI designed specifically for bereavement was evaluated (ie, effectiveness or feasibility), and (5) the study was an RCT or a feasibility study.

### Data Extraction

Data from each included study were extracted and collected independently by 2 investigators (M Luppa and CS). A standardized data extraction form was applied. The reliability of data abstraction was tested using a random sample. Discrepancies at each stage of the selection process were resolved by discussion with the inclusion of a third researcher (SGRH). The data extracted were study characteristics: author, year of publication, country, study design, sample sizes, response rates, and recruitment; participant characteristics: age and gender; methodological aspects: diagnostic approach, diagnostic criteria, inclusion and exclusion criteria, and measurements (effectiveness and feasibility); and intervention characteristics: name, description, duration, guidance, and focus. In addition, if necessary, the authors were contacted for further information.

### Quality Assessment

The risk of bias of the included studies was assessed by M Luppa and AEZ independently using the Cochrane Collaboration tool for assessing risk of bias [29]. The tool covers 6 domains of potential bias (eg, random assignment of participants to interventions, allocation concealment, and handling of missing data), with each domain labeled as *high*, *low*, or *unclear* for each study. The overall quality of evidence was assessed using the GRADE system [27].

A set of quality criteria suggested for IMIs by the DGPPN [1] was applied to assess the quality of the interventions described in the included studies. Quality criteria included information on therapeutic quality requirement, patient safety, information on mode of delivery (eg, guided or unguided), and data protection. These criteria were based on the *Model for Assessment of Telemedicine Applications* [30]. As these recommendations are aimed at already disseminated IMIs, the criteria were adapted slightly to be applicable to RCTs.

### Effect Sizes and Meta-Analytic Procedures

For all studies, effect sizes of changes in outcomes targeting symptoms of grief after bereavement between baseline or preintervention and postintervention (ie, treatment effect) were obtained from sample sizes, means, and SDs in the experimental and control groups of the trials. Effect sizes were included as between-group effect sizes per outcome using data from intention-to-treat analyses or per-protocol analyses in cases where intention-to-treat data were not available. Standardized mean group differences within the studies and a pooled overall effect size of a given outcome across studies were estimated using the Hedges method to adjust for heterogeneity in sample sizes [31]. This estimator can be interpreted similarly to Cohen *d*, whereby effect sizes <0.5 are considered small, 0.5-0.8 indicate a moderate effect size, and >0.8 indicate a strong effect size [32]. Heterogeneity was further inspected by applying *Q* and *I*^2^ statistics and forest plots. To account for diversity in trial outcomes focusing on grief treatment, stratified meta-analyses were run for the respective outcomes considered in the original studies. Funnel plots and Egger tests were applied to assess potential publication bias and small study effects. In addition, to identify potential determinants on the pooled estimates, meta-regression analyses were conducted including the variables dropout rate (intervention and control group), feedback from the therapist (binary variable, yes or no), number of sessions or assignments, time since loss, and age of participants. All analyses were conducted using Stata 16.0 (standard edition, StataCorp).

## Results

### Study Selection

**Figure 1:**
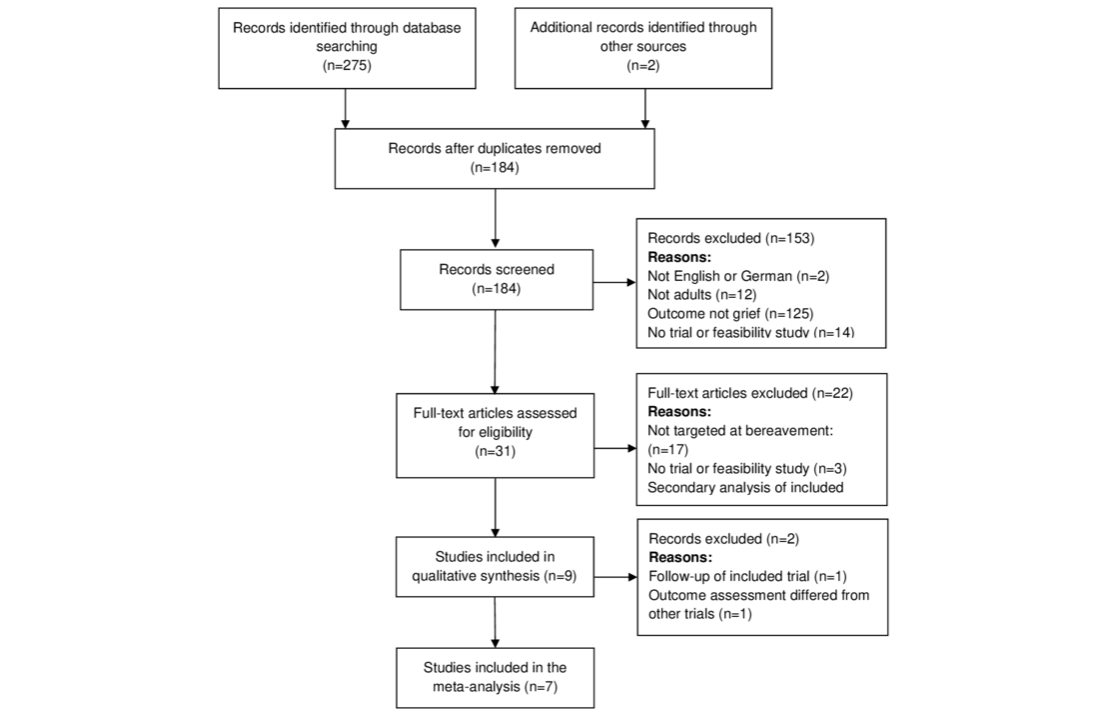
PRISMA (Preferred Reporting Items for Systematic Reviews and Meta-Analyses) flow diagram of the study selection process.

Of 275 studies identified through a literature search, 93 (33.8%) were duplicates and were therefore removed. After screening the titles and abstracts or reading the full text of the remaining articles, 4.9% (9/182) studies met the eligibility criteria and were included in the review. The selection process is illustrated in Figure 1. Most studies were excluded because the participants did not experience grief after bereavement or because the intervention did not address grief after bereavement (n=125).

### Description of Selected Articles

An overview of the characteristics of the study samples is provided in Multimedia Appendix 2 [33-41]*.* In total, 2 studies applied the same intervention [33,34,42] in different samples, whereas 1 study [35] tested 2 interventions (exposure and behavioral activation) against the same control group. Therefore, the number of interventions differed slightly from the number of included studies. The investigations of Wagner et al [36] and Wagner and Maercker [37] were based on the same population but reported data from different time points (posttreatment and 3-month follow-up and 1.5-year follow-up, respectively) and therefore were both included in the review.

All studies except 2 [34,37] were RCTs. In these studies, a simple randomization strategy [33,35-39] or a stratified block design [40,41] was used for randomization. The pilot study of Kersting et al [34] was nevertheless included because the results were compared with those of a randomized control group. Therefore, the study design can be regarded as an RCT. At baseline, there were no significant differences between the intervention and control groups in the 6 studies [34,36-40]. In 2 articles, there were significant differences between at most 2 measured scales [33,35]. In all, 1 study did not report differences between the intervention and control groups [41].

Most studies were implemented in German-speaking [33,34,36,37,41] or English-speaking [38-40] countries; 1 study [35] was conducted in the Netherlands. The sample sizes ranged from 25 [41] to 757 [39]. The samples mostly included women (range 67.9%-100%) and middle-aged adults (mean range 34.2-63.4, SD 5.2-7.8 years). Overall, the level of education was rather high in all included studies, as indicated by the large proportion of participants with a high level of education or a university or college degree.

Participants mainly reported the loss of a parent [38], relatives other than their partner (ie, child, sibling, or parent) [35], a child during pregnancy [33,34], a spouse [40,41], or a child [36,37,39]. Certain trials were designed for specific types of death (eg, expected loss as a result of natural death [38] and prenatal loss of a child [33,34,42]), whereas the remaining trials were not restricted in this regard. However, it must be noted again that the intervention applied in the study by Kersting et al [34] was the same as in the study by Kersting et al [33], whereas the study by Wagner and Maercker [37] displayed the follow-up data from the study by Wagner et al [36]. The time since loss varied considerably among the trials, ranging from 1 to 6 months [38] to several years [36,39].

A description of the study characteristics is provided in Multimedia Appendix 3 [33-41]. All interventions were web-based and delivered as individual therapy. No study tested a mobile-based program. Most studies focused on complicated grief [33,34,36,37], whereas others focused on normal grief [38], complicated grief and rumination [35], prolonged grief [40,41], and bereavement [39]. For reasons of simplicity and because of similar eligibility criteria of the included articles, these terms are summarized as *grief*. Furthermore, 6 studies assessed posttraumatic stress symptoms (PTSS) [33-37,40] and 8 studies assessed depressive symptoms [33-37,39-41].

The duration of treatment ranged from 2 days [38] to 3 months [39], whereas most interventions lasted 5 weeks [33,34,36,37]. In total, 8 studies used a wait-list control group design. In another RCT, the researchers applied a treatment-as-usual control group [38]. Attrition rates ranged from 0% [38] to 59% [39].

Descriptions of the interventions are presented in Table 1. Except for *Making Sense of Grief* [38], which is a psychoeducational self-help tool based on social cognitive theory, all interventions were based on elements of CBT. With the exception of the study by Van der Houwen et al [39], all CBT-based interventions included distinct modules on exposure and cognitive reappraisal. In total, 2 interventions [35,40] included elements of behavioral activation.

**Table 1:** Description of the interventions.

Study	Therapeutic approach	Intervention components	Exposure	Cognitive reappraisal	Behavioral activation	Therapist feedback
Brodbeck et al [41]	CBT^a^	Text-based modules including writing assignments, covering the areas psychoeducation, assessment of current situation, fostering positive thoughts and emotions, finding comfort, self-care, and accepting memories	Yes	Yes	No	Yes
Dominick et al [38]	Social cognitive theory	3 intervention modules (“My grieving style”; “Who am I?”; and “How am I doing?”), including interactive exercises supplemented by video testimonials; type-in responses and check lists; additional models: “Grief experience” and “Resources” offering text articles and websites or books covering grief-related topics	No	Yes	No	No
Eisma et al [35]	CBT	Email-based homework assignments; exposure condition: writing assignments, imaginal or in vivo exposure exercises; behavioral activation condition: 7-day activity diary, identification of pleasurable and meaningful activities, identification of personal core values, development of new meaningful and pleasurable activities based on these values	Yes	No	Yes	Yes
Van der Houwen et al [39]	CBT	Email-based writing assignments; exposure: describing the most distressing aspects of the loss (2 assignments); cognitive reappraisal: information on and identification of dysfunctional grief cognitions, letter to hypothetical bereaved friend (2 assignments); integration or restoration: letter to the deceased (1 assignment)	Yes	Yes	No	No
Litz et al [40]	CBT	Internet-based psychoeducation (18 sessions); education about loss and grief, instruction on stress management and other coping skills, behavioral activation: assignments on self-care and social re-engagement, accommodation of loss by establishing and working toward a personalized goal, and relapse prevention	No	No	Yes	Yes
Kersting et al [34]	CBT	Email-based writing assignments; self-confrontation: describing the circumstances of the loss (4 assignments); cognitive restructuring: supportive letter to hypothetical bereaved friend (4 assignments); social sharing: symbolic farewell letter to oneself, a loved one, or a person connected to the loss (2 assignments)	Yes	Yes	No	Yes
Kersting et al [33]	CBT	Similar intervention components as Kersting et al [34]	Yes	Yes	No	Yes
Wagner et al [36]	CBT	Email-based writing assignments; exposure: describing the circumstances of the loss, specifically distressing loss-related thoughts (4 assignments); cognitive reappraisal: letter to hypothetical bereaved friend, identification of new role or identity after the loss and possible rituals to remember the deceased by, activation of social resources and competencies (4 assignments); integration and restoration: outlining important memories about the loss; reflecting on therapeutic process and grieving style; letter to oneself, a significant person, or a person related to the loss	Yes	Yes	No	Yes
Wagner and Maercker [37]	CBT	Similar intervention components as Wagner et al [36]	Yes	Yes	No	Yes

^a^CBT: cognitive behavioral therapy.

An unguided internet-based treatment was applied in 2 studies [38,39]. All other interventions were guided via email [33-36,41] or telephone [40]. Guidance involved individual written feedback [33,34,36,37,42], technical information on how to use the intervention [35,39], or technical assistance via email or telephone and short reminders for participants with longer periods of inactivity [40]. Most interventions included writing assignments dealing with specific aspects of the loss [35,41] and exposure condition [33,34,36,37,39,42].

### Effect Sizes

**Figure 2:**
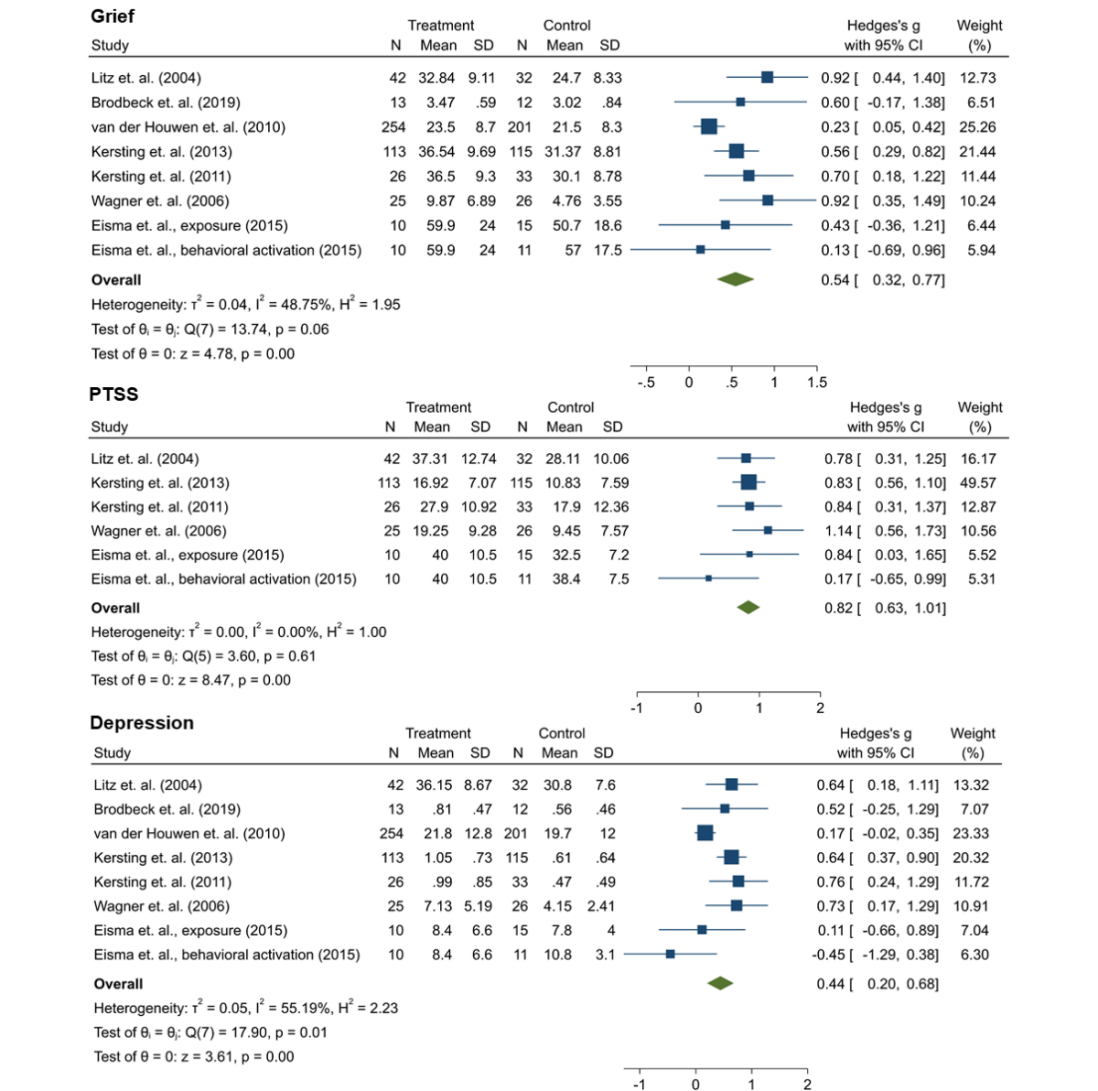
Effect sizes of interventions for grief, posttraumatic stress symptoms, and depression [33,34,36,37,40-42]. PTSS: posttraumatic stress symptoms.

The forest plots of between-group effect sizes at the postintervention assessment for grief, PTSS, and depression across the studies are shown in Figure 2. Effect sizes ranged from moderate (grief: *g*=0.54, 95% CI 0.32-0.77; depression: *g*=0.44, 95% CI 0.20-0.68) to large (PTSS: *g*=0.82, 95% CI 0.63-1.01), whereas heterogeneity was low for PTSS (*I*^2^=0%) and moderate for grief (*I*^2^=48.75%) and depression (*I*^2^=55.19%). In total, 2 studies were excluded from the meta-analysis because they covered outcomes other than grief, depression, or PTSS [38] or follow-up data from included samples [37]. The results of the meta-regression analyses for grief and depression revealed that none of the considered determinants was associated with the respective pooled effect sizes. The Egger test revealed no indication of small study bias for grief (*P*=.16), PTSS (*P*=.62), or depression (*P*=.62). Funnel plots indicated the presence of publication bias for grief and depression. Meta-regression results and contour-enhanced funnel plots are provided in Multimedia Appendix 4.

### Feasibility

Satisfaction with the internet-based intervention or other aspects of feasibility was measured in 5 studies [35,36,38,40,41], and their respective measures and results are provided in Table 2. Dominick et al [38] assessed acceptability and usability using items derived from web evaluation instruments, whereas Brodbeck et al [41] assessed satisfaction with self-constructed items derived from a validated questionnaire on patient satisfaction [43]. The trial by Litz et al [40] relied on the Post-Study System Usability Questionnaire and a protocol evaluation questionnaire, whereas Eisma et al [35] used a standardized questionnaire derived from earlier interventions for bereavement [44].

**Table 2:** Feasibility and satisfaction with treatment.

Study	Outcome assessment	Rating^a^
Brodbeck et al [41]	11 items measuring satisfaction; 4-point scale (1=not at all to 4=very much)	3.36 (0.32)
Dominick et al [38]	4 items measuring satisfaction (usefulness, helpfulness, satisfaction with the intervention, and recommendation to friends; 7-point Likert scale, 1=not at all to 7=extremely); 6 items measuring usability and acceptability (6-point Likert scale, 1=strongly disagree to 6=strongly agree); open question on possibilities to improve intervention	Satisfaction: satisfied with the intervention, 5.18 (1.47); recommendation, 5.62 (1.52); helpful for understanding grief, 5.15 (1.54); useful for coping with grief, 4.85 (1.35) Acceptability or usability: interesting, 4.88 (0.91); easy to use, 5.21 (0.81); attractive, 5.00 (0.82); liked guidance and structure, 5.21 (0.84); videos believable, 5.03 (0.87); videos add to value of intervention, 5.12 (0.91)
Eisma et al [35]	6 items measuring feasibility (comprehensibility of instructions and homework, feeling understood by the therapist, general feasibility, usefulness of treatment, and satisfaction with treatment), 5-point scale (1=completely disagree to 5=completely agree)	Exposure: comprehensibility of instructions/homework, 4.67 (0.60)/4.67 (0.48); feeling understood by the therapist, 4.36 (0.63); general feasibility, 4.21 (1.05); usefulness of treatment, 4.00 (1.17); satisfaction with treatment, 3.86 (0.95) Behavioral application: comprehensibility of study information/homework assignments, 4.64 (0.51)/4.27 (0.78); feeling understood by therapist, 4.13 (0.94); general feasibility, 3.64 (1.21); usefulness of treatment, 3.64 (1.21); satisfaction with treatment, 3.64 (1.21)
Litz et al [40]	Acceptability or feasibility (PSSUQ^b^; 13-item 7-point scale, 1=strongly agree to 7=strongly disagree); system usefulness: ease, simplicity, efficiency of learning to use the website and using the website; information quality: is the information on the use of the website clear, easy to understand, and effective for helping with completion of the tasks?; protocol evaluation questionnaire: personal relevance and meaningfulness of intervention modules, accessibility of information, and general reactions to the intervention and its web-based format; qualitative feedback on intervention	PSSUQ usefulness subscore, 3.02 (2.16); PSSUQ information quality subscore, 2.95 (2.06) Protocol evaluation questionnaire: content was logical, 7.16 (1.7), best possible value: 9; amount of information: 6% “somewhat too much”, 77.6% “just the right amount”, 16.4% “would have preferred more information”; instruction level: 77.6% “just right”, 20.9% “somewhat too basic”, 1.5% “far too basic”; satisfaction with content: 53.7% learned a moderate amount, 35.8% learned a large amount from the program; interest: 43.3% “extremely interesting”, 53.7% “somewhat interesting”; individual components: >90% consistently rated modules “moderately valuable” to “extremely valuable”; likelihood of recommendation: 7.37 (1.9), best possible value: 9
Wagner et al [36]	4 items measuring treatment experience: contact with therapist (personal, impersonal, or do not know), experience of therapist contact via email (unpleasant, pleasant, or do not know), missing face-to-face contact with therapist (no, yes, or I do not know), and assumed effectiveness of intervention to reduce complaints (no, a little, quite a bit, or very strongly)	Therapist contact via email: 85% (“pleasant”); missing face-to-face-communication (”yes“): 20%; contact with therapist: 83% (”personal“); effectiveness: 45% (”quite a bit“); 10% (”very strongly“)

^a^Results reported as mean (SD) or percentage.

^b^PSSUQ: Post-Study System Usability Questionnaire.

### Methodological Quality

#### Risk of Bias

**Figure 3:**
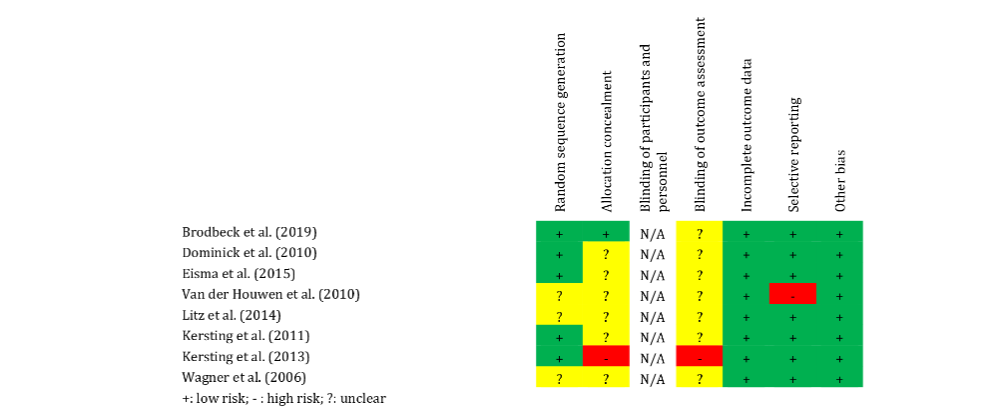
Risk of bias in included randomized controlled trials based on Higgins et al [29].

The risk of bias was assessed using the Cochrane Collaboration tool for assessing risk of bias [29], indicating low or high risk of bias for each study across 6 domains (Figure 3). Risk in a specific domain was labeled *unclear* if sufficient information was not available. As blinding of participants is not feasible in intervention trials requiring active participation and most trials included at least some kind of feedback from therapists or other study personnel, the domain blinding of participants and *personnel* was labeled *not applicable* for all trials. The risk of bias assessment for the individual studies is provided in Multimedia Appendix 5 [33-41].

#### Overall Quality of Evidence

The quality of evidence, assessed using the GRADE criteria, was considered low for depression and moderate for grief and PTSS. The domains of quality assessment for the 3 outcomes are presented in Table 3.

**Table 3:** Quality of evidence across studies (Grading of Recommendations, Assessment, Development, and Evaluations [27]; n=8).

Quality assessment		Outcome measure		
		Grief (n=8)	Depression (n=8)	PTSS^a^ (n=6)
**Downgrade in quality of evidence**				
	Risk of bias	No	No	No
	Inconsistency	No^b^	No^b^	No^b^
	Indirectness	No	No	No
	Imprecision	Yes	Yes	Yes
	Publication bias	Suspected^c^	Suspected^c^	Suspected
**Upgrade in quality of evidence**				
	Large effect	No	No	Yes
	Possible confounding would change effect	No	No	No
	Dose-response effect	No	No	No
	Effect (95% CI)	0.54 (0.32-0.77)	0.44 (0.20-0.68)	0.82 (0.63-1.01)
Overall quality of evidence		Low	Low	Moderate

^a^PTSS: posttraumatic stress symptoms.

^b^*I*^2^<60%.

^c^As indicated by funnel plots.

#### Quality Criteria for IMIs

In addition to the methodological quality of the studies, we assessed the quality of the interventions described in the included studies based on recommendations by the DGPPN adapted for RCTs. The results are presented in Table 4. We also included an item covering information on potential funding sources and their role in the conduction of the study. If information on the intended purpose of the intervention was not available on the web and could not be obtained from the corresponding study authors, the criterion was marked as *unclear*. The overall quality varied across the interventions, whereas 2 interventions met all 12 criteria [34,41]. The quality of other interventions ranged from 5 [38] to 10 points [40].

**Table 4:** Quality assessment of internet- and mobile-based interventions.

Item		Study						
		Brodbeck et al [41]	Dominick et al [38]	Eisma et al [35]	Van der Houwen et al [39]	Litz et al [40]	Kersting et al [33,34]	Wagner et al [36]
**Indication**								
	Is the intended purpose of the intervention clearly stated (which psychological symptoms can be alleviated by the intervention, orientation toward current version of the ICD^a^ and empirical evidence regarding the intervention)?	Yes	Unclear	Unclear	Unclear	Unclear	Yes	Unclear
**Description of the intervention**								
	Is the intervention based on evidence-based theories and techniques of psychotherapy? Are these theories and techniques clearly stated?	Yes	Yes	Yes	Yes	Yes	Yes	Yes
	Information whether intervention is guided or unguided	Yes	No	Yes	Yes	Yes	Yes	Yes
	If guided, is there information on the type and content of guidance and who initiates contact?	Yes	N/A^b^	Yes	N/A	Yes	Yes	Yes
	Information on how often or how frequently the intervention should be used, possible prerequisites	Yes	No	Yes	Yes	Yes	Yes	Yes
**Qualification**								
	Was the intervention developed by registered psychotherapists or specialists in the field of psychiatry, psychotherapy, or psychosomatic medicine or affected parties? Is their possible involvement clearly stated?	Yes	Yes	Yes	Yes	Yes	Yes	Yes
	Exclusion of participants with full-blown disorders (eg, severe depression and suicidal ideation)	Yes	Yes	Yes	Yes	Yes	Yes	Yes
**Effectiveness**								
	Use of intention-to-treat analyses to estimate effects	Yes	No	Yes	Yes	Yes	Yes	Yes
	Between-group Cohen *d* is reported for primary outcome (determined in advance)	Yes	No	Yes	Yes	Yes	Yes	Yes
	Has the trial been registered in a clinical trial register?	Yes	No	No	No	Yes	Yes	No
**Safety of patients**								
	Advice on handling of crises (eg, referring to professional care with face-to-face contact); if people with full-blown disorders are included: assessment of emergencies and immediate reference to professional help	Yes	Yes	No	No	No	Yes	No
	Provision of information on potential funding sources and their role in the conduction of the study	Yes	Yes	Yes	No	Yes	Yes	No
Number of criteria^c^ fulfilled (n=12), n (%)		12 (100)	5 (42)	9 (75)	7 (58)	10 (83)	12 (100)	8 (67)

^a^ICD: International Statistical Classification of Diseases and Related Health Problems.

^b^N/A: not applicable.

^c^Quality criteria based on the study by Klein et al [1].

## Discussion

### Principal Findings

This review systematically assessed the effectiveness of IMIs in treating symptoms of grief, depression, or PTSS after bereavement. We also provided information on the feasibility and quality of the delivered interventions based on quality criteria proposed by a professional organization in the field of mental health, namely, the DGPPN. Internet- or mobile-based interventions for grief after bereavement were found to be effective against symptoms of grief, PTSS, and depression, with the largest effect sizes observed for PTSS. These findings are in line with a recent review by Wagner et al [45] and Johannsen et al [19] covering face-to-face interventions targeting grief symptoms; however, the observed effects were lower than the effect sizes reported for IMIs targeting anxiety disorders, depression, or insomnia (for a meta-review, please see Stein et al [10]).

The observed treatment effects were smaller for depression (*g*=0.44) than for grief symptoms (*g*=0.54), which might indicate differences in symptomatology between grief and depression. Recent network analyses have found symptoms such as disturbed sleep, fatigue, anhedonia, and psychomotor agitation to be characteristic of major depressive disorders but not of persistent complex bereavement disorder [46]. It is possible that the interventions tested in the included studies are more suitable for addressing symptoms of grief than symptoms of depression. Addressing the latter in future interventions targeting bereaved individuals could further improve symptoms of depression. Most included studies relied on email-based writing assignments as part of the treatment; other IMIs specifically targeting depression included animated demonstrations or focused on increasing physical and social activity [47]. The individual intervention components should be tested in future trials.

The largest effect sizes were observed for PTSS. Regarding individual studies, however, the strongest effects for PTSS were observed in trials specifically addressing parents or women who had lost a child [33,34] or comprised samples where most participants had experienced the loss of a child [36]. Several studies reported pregnancy loss or loss of a child to be a risk factor for PTSD [48,49], and a review on face-to-face grief counseling identified parents mourning the loss of a child as *high-risk mourners* [50]. These factors might have led to a high proportion of traumatic loss experiences in the analyzed samples, contributing to the observed large effect size for PTSS.

Except for the study by Van der Houwen et al [39], all studies included in the meta-analysis applied guided interventions; therefore, current evidence is strongest for IMIs including a predetermined type of contact between the patient and therapist. This might point toward a useful treatment option for patients currently unwilling or unable to seek face-to-face mental health care or to discuss problems related to grief. On the other hand, IMIs could be integrated into regular care of patients experiencing grief after bereavement, and future trials are needed to provide more information on the potential of unguided interventions.

Assessment of the included interventions revealed high levels of quality, that is, instructions on how and how often to use the intervention, information on type of guidance by psychologists or other study personnel, and advice on handling of acute crises were provided. However, only a limited number of trials testing the interventions had been registered in a clinical trial register. All but 2 interventions [34,41] had no active address on the web at the time of the review or information of the presence of the intervention on the web could not be obtained retrospectively; therefore, certain aspects (eg, information on indication and purpose of the intervention provided for participants) could not be evaluated for all studies.

Furthermore, 5 (56%) out of 9 studies assessed feasibility or user satisfaction [35,36,38,40,41], revealing moderate to high levels of user satisfaction on average. Most participants regarded the interventions as both understandable and helpful. However, not all studies systematically assessed aspects of feasibility. Additional aspects could be covered in future trials, for example, time needed to complete the intervention or the intervention components on the part of the participants; certain studies included in this review reported considerable differences between scheduled and actual time needed to complete the intervention [40]. In addition, information on the amount of time devoted to feedback on assignments or inquiries from participants by psychologists or study personnel could provide useful information on the cost-effectiveness of the respective interventions [11]. The overall quality of evidence, as assessed by the GRADE criteria, was rated low for grief and depression and moderate for PTSS, particularly because of wide CIs and the possibility of publication bias.

In addition to the observed positive effects of IMIs against symptoms of grief, depression, and PTSS, future studies are needed to investigate the underlying mechanisms of the effects of these treatments (ie, what makes internet- or mobile-based approaches effective [19,34]). This should also include a more detailed feasibility assessment of the respective intervention components (eg, psychoeducation, exposure, behavioral activation, and therapist feedback) to investigate which components provide the most beneficial effect against the respective symptoms. Most included studies applied a wait-list control design; future research investigating different settings and study designs (eg, combined use of IMI and face-to-face CBT or evaluation against another mental health IMI) could yield valuable results on the effectiveness of IMIs against grief symptoms. Although the studies discussed in this review relied on self-reported data on symptoms of grief, PTSS, and depression, further investigations using clinical interviews to assess change in symptom load and symptom severity could further elucidate our knowledge on the effectiveness of IMIs. Respective analyses could yield valuable information as, in a systematic review, the effectiveness of face-to-face interventions was found to be related to symptom severity at baseline [22].

### Limitations

Certain limitations need to be pointed out when interpreting our findings. Most participants in the included studies were women; therefore, we can only make limited assumptions about the effectiveness of the treatments for men. Recent reviews and meta-analyses have reported that men and women are equally affected by prolonged grief following natural or unnatural losses [20,51], highlighting the need for interventions for both men and women. Although some interventions were specifically targeted at women (eg, interventions aimed at grief after pregnancy loss), the effectiveness of IMIs for grief after bereavement in men remains an unsettled question. Beyond that, the level of education was comparatively high in the included studies, possibly indicating selection bias. Future trials might consider a wider variety of recruitment strategies to achieve more gender-balanced samples and a greater diversity of education levels, possibly increasing the generalizability of the results. Furthermore, this review and meta-analysis relied on a relatively small number of studies with partially very small sample sizes, stressing the need for further RCTs assessing the effectiveness of IMIs for grief after bereavement.

### Conclusions

Our review provides evidence for the potential of IMIs as a safe and effective approach for treating symptoms of grief, depression, and posttraumatic stress after bereavement. Owing to the low cost and high accessibility, IMIs could benefit a large number of individuals experiencing grief after the loss of a significant other. With the inclusion of conditions such as persistent complex bereavement disorder or prolonged grief disorder in the Diagnostic and Statistical Manual of Mental Disorders and the International Statistical Classification of Diseases and Related Health Problems, awareness of the potential of IMIs targeting grief after bereavement should be raised among clinicians and decision makers in mental health care.

The proof of effectiveness provided by RCTs is a central prerequisite for the implementation of new treatments in health care systems. The evidence reported in this review might therefore contribute to the advancement of IMIs for grief in bereaved individuals and their certification and implementation in routine care in the future. Further studies are warranted to deepen our knowledge on what makes IMIs successful for which populations of bereaved individuals and on the needs and preferences of users. This could contribute to improved care for and well-being of those experiencing grief after bereavement.

## Appendix

Multimedia Appendix 1 Search strategy.

Multimedia Appendix 2 Description of study samples.

Multimedia Appendix 3 Study characteristics.

Multimedia Appendix 4 Meta-regression analysis for grief, posttraumatic stress symptoms, and depression.

Multimedia Appendix 5 Risk of bias assessment for individual included studies.

## Abbreviations

CBT: cognitive behavioral therapy
DGPPN: Deutsche Gesellschaft für Psychiatrie und Psychotherapie, Psychosomatik und Nervenheilkunde (German Association for Psychiatry, Psychotherapy, and Pschosomatics)
GRADE: Grading of Recommendations, Assessment, Development, and Evaluations
IMI: internet- and mobile-based intervention
PRISMA: Preferred Reporting Items for Systematic Reviews and Meta-Analyses
PTSS: posttraumatic stress symptoms
RCT: randomized controlled trial
