# Supplementary material for: Effectiveness and Feasibility of Internet-Based Interventions for Grief After Bereavement: Systematic Review and Meta-analysis
Source: JMIR Ment Health. 2021 Dec 8;8(12):e29661. doi: 10.2196/29661 (PMC8701663; doi:10.2196/29661)

**Search strategy**

**PubMed** (last search: 09^th^ January 2020)


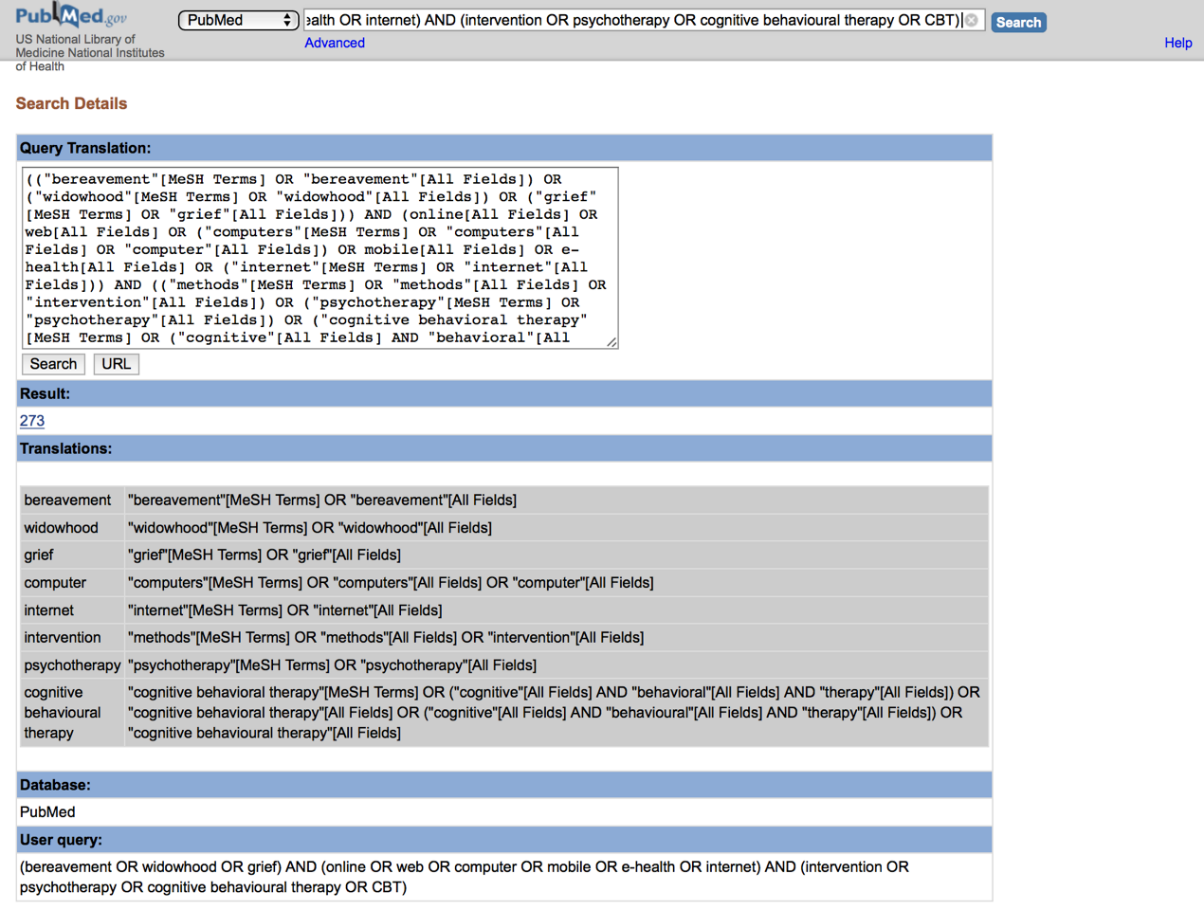


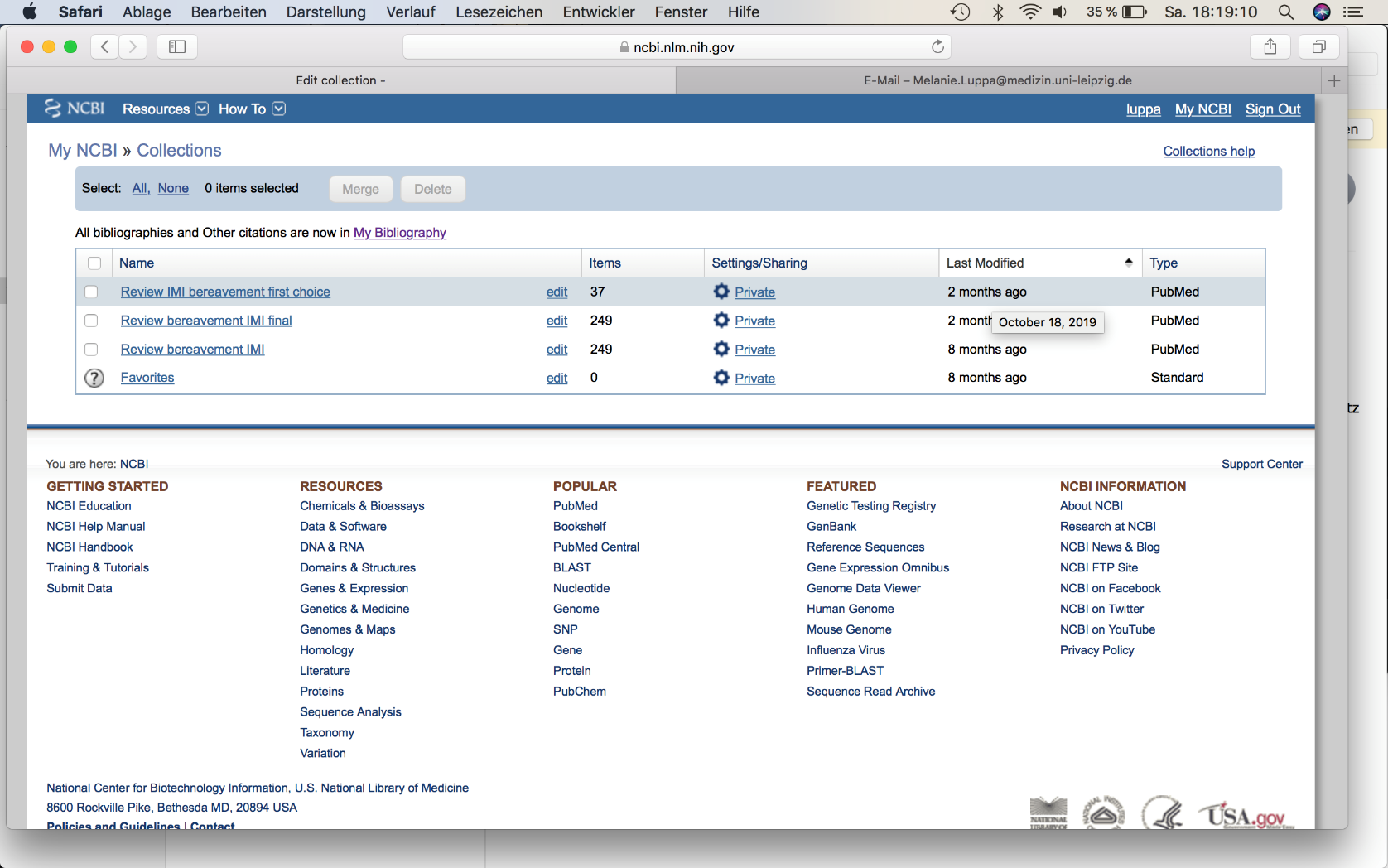


**Cochrane Central Register of Controlled Trials** (last search: 12^th^ January 2020)


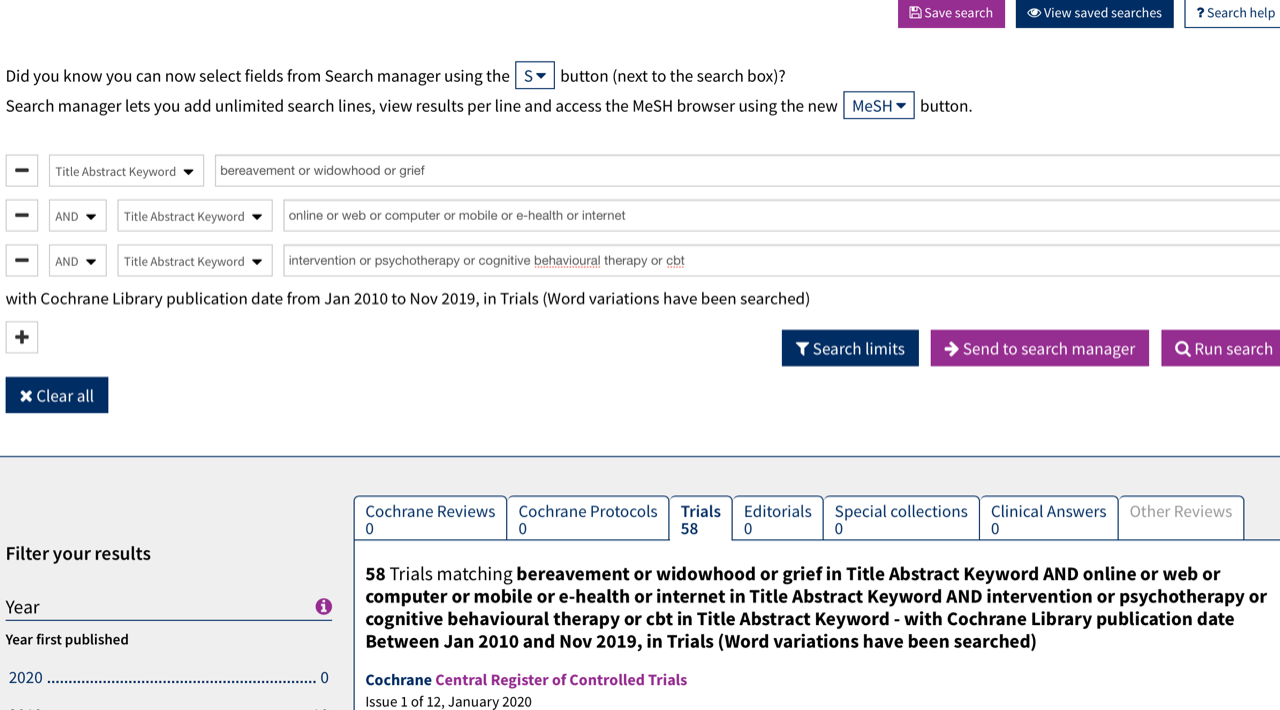

Supplement: Multimedia Appendix 1 [file mental_v8i12e29661_app1.docx]
