# Supplementary material for: Effectiveness and Feasibility of Internet-Based Interventions for Grief After Bereavement: Systematic Review and Meta-analysis
Source: JMIR Ment Health. 2021 Dec 8;8(12):e29661. doi: 10.2196/29661 (PMC8701663; doi:10.2196/29661)
Supplement: Multimedia Appendix 2 [file mental_v8i12e29661_app2.docx]

**Description of study samples**

| Study | Country | Eligibility criteria | Exclusion criteria | Population; recruitment strategy | Age, mean (SD) [range] | Female, % | Education, % | Relation to deceased | Type of loss | Time since loss, mean (SD), [range] in months |
| --- | --- | --- | --- | --- | --- | --- | --- | --- | --- | --- |
|  |  |  |  |  |  |  |  |  |  |  |
| **Brodbeck et al. 2019 [41]** | Switzerland | experiencing spousal bereavement > 6 months before enrolment; seeking help for coping with loss; internet access; ability to speak German; informed consent | severe psychological or somatic disorders, acute suicidality; no emergency plan for acute crises; concomitant psychotherapy, and /or prescribed drugs against depression: inability to follow instructions of the study | population-based study; newspaper articles, internet-self-help forums | 63.4 (7.8) | 80 | Apprenticeship: 24; Secondary: 8; Technical college: 44; University: 24 | Spouse | Not stated | 25.08 (20.76) |
| **Dominick et al. 2010 [38]** | USA | Experiencing bereavement 1-6 months ago (natural death of parent/older relative), uncomplicated grief, ≥ 18 years of age, living in the United States, computer- and e-mail access for duration of study | Symptoms of depression, urgent/immediate need of care | population-based study; grief support websites, listservs, online message boards, Internet advertising, newsletters, e-mail announcements, e-newsletters, newspaper-ads | 47 (12) | 87 | Some high school: 3.0;  High school graduate: 10.4; Some college: 32.8; College graduate: 20.9; Graduate/professional: 29.9; Trade school: 3.0 | Parent or older relative | Natural death | [1-6 months] |
| **Eisma et al. 2015 [35]** | Netherlands | experiencing bereavement of 1^st^ degree relative > 6 months before enrolment; elevated levels of complicated grief, elevated grief rumination; ability to understand Dutch, access to computer, basic computer skills | current suicidal plans, past or current psychosis or schizophrenia, past or current episodes of dissociation or dissociative disorder | population-based study; online advertisements (websites, Facebook pages of organizations for bereaved individuals), content network of Google | 46 (13) | 92 | Lower education: 40.4; Higher education: 59.6 | Partner: 40.4%; child, sibling, parent: 59.6% | Non-violent: 78.7%; violent: 21.3% | 31.0 (45.1) |
| **Van der Houwen et al. 2010 [39]** | UK, USA | ≥ 18 years of age, native English speaker, having experienced death of a first-degree relative, still being significantly distressed by this loss | Self-reported severe depression, schizophrenia, psychotic episodes, suicidal ideation; people who suffered their loss at a very early age. | population-based study; through websites, forums, and e-mail groups that focus on bereaved persons; via organizations and support groups for the bereaved | 43.2 (10.98) [18–81] | 93.5 | Low: 16.8; Medium: 47.0 High: 36.2 | Child: 42.5%; partner: 30.4%; parent: 16.6%;, sibling: 10.4% | Natural: 65.8%; accident/homicide: 22.1%; suicide: 12.2% | 40.44 (62.88) |
| **Litz et al. 2014 [40]** | USA | ≥ 21 years of age experiencing bereavement 3-6 months before enrolment; elevated symptoms of prolonged grief disorder, functional impairment functional impairment in social, occupational, or household responsibilities; internet access | schizophrenia, delusional disorders, substance abuse/dependence in the last year; current suicidality, current participation in bereavement support groups; inability to understand study procedures or provide informed consent | population-based study; bereaved caregivers of recently deceased patients who had been treated at the Dana-Farber Cancer Institute in Boston, Massachusetts. | 55.37 (10.30) | 68 | some high school: 1.2; High school diploma: 7.1; Some college: 17.9; associates degree: 6.0; 4-year college degree: 28.6; masters degree: 32.1; doctoral degree: 7.1 | spouse: 76.2%; partner: 6.0%; child: 4.8%; parent: 7.1%; sibling: 2.4%; relative: 2.4%; friend: 1.2% | Not stated | 8.38 (2.97) |
| **Kersting et al. 2011 [34]** | Germany | Mothers experiencing prenatal death of a child; living in a German-speaking country, being a German native speaker, internet access, written informed consent | Minors, existing pregnancy at allocation, severe depression, suicidal tendencies, dissociative tendencies, risk of psychosis, substance abuse/dependence, psychotherapy at allocation | population-based study; advertisements in newspapers, internet pages, own website, distribution of flyers in five cooperating centers and in associated clinics and medical surgeries | 34.3 (5.34) | 100 | Low: 9.0;  Medium: 44.9; High: 46.2 | Child | Early miscarriage: 51.3%; late miscarriage: 16.7%; medically indicated abortion: 12.8%; stillbirth: 19.2% | 15.4 (27.4) [1-144] |
| **Kersting et al. 2013 [33]** | Germany | parents experiencing prenatal death of a child; ≥ 18 years of age, living in a German-speaking European country, fluency in German, internet access, written informed consent | severely depressed mood or suicidal ideation, dissociative tendencies, risk of psychosis, current pregnancy, substance abuse and dependency, currently receiving treatment elsewhere | population-based study; self-referral via study website | 34.18 (5.15) | 92.1 | Low: 4.4;  Medium: 12.7; High: 82.9 | child | prenatal death: 100% | 9.93 (24.11) |
| **Wagner et al. 2006 [36]** | Germany, Austria, Switzerland, other (German native speakers living abroad) | symptoms of intrusion, avoidance, maladaptive behavior caused by the death of a significant other | Currently receiving other treatment, substance abuse, loss < 14 months ago, age < 18 years, not fluent in German, severely depressed mood, suicidal ideation, dissociative tendency, risk of psychosis | population-based study; announcements in print media, advertisements/links on homepages of organizations for bereaved persons and psychology websites | 37.9 (10.2) [19-68] | 94.4 | Low: 15.7;  Medium: 39.2,  High: 31.4 | child :61%; partner/spouse: 10%; sibling: 12%; parent(s): 6%; relative: 4%; friend: 4% | Illness: 35%; accident: 27%; homicide/suicide: 18%; stillbirth/sudden infant death: 17% | 55.2 (79.2) [14-348] |
| **Wagner & Maercker 2007 [37]** | Germany, Austria, Switzerland, other (German native speakers living abroad) | symptoms of intrusion, avoidance, maladaptive behavior caused by the death of a significant other | Currently receiving other treatment, substance abuse, loss < 14 months ago, age < 18 years, not fluent in German, severely depressed mood, suicidal ideation, dissociative tendency, risk of psychosis | 1.5-year follow-up data from [37] | 36 (11) [18-68] | 88 | See [37]. | child: 61%; partner/spouse: 10%; sibling: 3%; parent(s): 10%; relative or friend: 16% | illness: 42%; accident: 23%; homicide/suicide: 19%; stillbirth/sudden infant death: 16% | 48 (60) [14-192] |
| SD: standard deviation | | | | | | | | | | |
