# Supplementary material for: Effectiveness and Feasibility of Internet-Based Interventions for Grief After Bereavement: Systematic Review and Meta-analysis
Source: JMIR Ment Health. 2021 Dec 8;8(12):e29661. doi: 10.2196/29661 (PMC8701663; doi:10.2196/29661)
Supplement: Multimedia Appendix 3 [file mental_v8i12e29661_app3.docx]

**Study characteristics**

| Study | Treatment condition | Outcome | Measurement of primary outcomes / secondary outcomes | N of sessions / duration (post, follow-up) | Sample size: Intervention group (randomization / post / FU) // waitlist control (randomization / post / FU) | Dropout rate IG / CG |
| --- | --- | --- | --- | --- | --- | --- |
| **Brodbeck et al. 2019 [41]** | Exposure / waitlist control | Grief, depression, psychopathological distress (primary outcomes); embitterment, loneliness, life satisfaction (secondary outcomes) | TRIG-D (grief), BDI-II (depression), BSI (general psychopathological distress); Short version of Embitterment Scale (embitterment), De Jong Gierveld Short Scale for Emotional and Social Loneliness (loneliness), SWLS (life satisfaction) | 10 sessions, 12 weeks | 12 // 13 | 12.0% |
| **Dominick et al. 2010 [38]** | Exposure / control | Attitude, self-efficacy, state anxiety | Self-constructed items (n=19 addressing attitude, n = 3 assessing self-efficacy), 6-point Likert-scale, 1 (“Strongly Disagree”) - 6 (“Strongly Agree”); STAI (anxiety) | 2 days | 33 // 34 | 0 |
| **Eisma et al. 2015 [35]** | Exposure / waitlist control | Complicated grief, grief rumination, depression, anxiety, posttraumatic stress, depressive rumination | ICG-R (complicated grief); PSS (PTSD); HADS (depressive symptoms, anxiety); UGRS (grief rumination); RRS (depressive rumination) | 6 assignments; 6-8 weeks, 3 months | EX: 18 / 15 / 12 // BA:17 / 11 / 11 // 12 / 10 / 10 | EX: 6 (33.3); BA: 10 (58.8) // 2 (16.7) |
| **Van der Houwen et al. 2010 [39]** | Exposure / waitlist control | Grief reactions, depressive symptoms, positive mood, emotional loneliness | 9 items based on criteria for complicated grief proposed for DSM-V (grief reactions); CES-D (depressive symptoms); PANAS (positive mood); 2 items based on [43] (emotional loneliness) | 5 assignments / 3 months, 6 months | 460 / 297 | 59% // 27% |
| **Litz et al. 2014 [40]** | Exposure / waitlist control | Prolonged grief (primary outcome); depressive symptoms, arousal and anxiety symptoms, posttraumatic stress symptoms, alcohol- and drug abuse | PG-13 (prolonged grief); BDI-II (depressive symptoms), BAI (arousal and anxiety), PCL-C (posttraumatic stress), AUDIT (alcohol- and drug abuse) | 18 sessions, 6 weeks, 3 months FU | 41 / 32 / 31 / 31 // 43 / 42 / 35 / 35 | 22.0 % // 2.3% |
| **Kersting et al. 2011 [34]** | Exposure / waitlist control | Posttraumatic stress, grief, depression, general psychopathology, anxiety, somatization | IES (traumatic stress), ICG (grief), BSI (general psychopathology, depression) | 10 assignments, 5 weeks, 3 months FU | 45 // 33 | 26.67 % //21.21% |
| **Kersting et al. 2013 [33]** | Exposure / waitlist control | PTSD, prolonged grief, general psychopathology (depression, anxiety) | IES (traumatic stress), ICG (grief), BSI (general psychopathology, depression) | 10 assignments, 5 weeks, 3 months FU | 115 / 99 / 85 / 45; 113 / 100 | 13.91% // 11.5% |
| **Wagner et al. 2006 [36]** | Exposure / waitlist control | intrusion, avoidance, failure to adapt; depression, anxiety, general mental / physical health | IES (intrusion, avoidance), symptom list for complicated grief (failure to adapt), BSI (depression, anxiety), SF-12 (general mental / physical health) | 5 weeks | 29 / 26 / 25; 26 / 25 | 10.3% // 3,8% |
| **Wagner & Maercker 2007 [37]** | Exposure / waitlist control | intrusion, avoidance, failure to adapt; depression, anxiety, general mental / physical health | IES (intrusion, avoidance), symptom list for complicated grief (failure to adapt), BSI (depression, anxiety), SF-12 (general mental / physical health) | 3 months FU | 26 / 22; 25 (CG did not receive FU assessment) | 15.4% |
| AUDIT: Alcohol Use Disorder Identification Test [44]; BA: behavioral activation; BAI: Beck Anxiety Inventory [45]; BDI-II: Beck Depression Inventory II [46]; BSI: Brief Symptom Inventory [47]; CES-D: Center for Epidemiological Studies-Depression Scale [48]; CG: Control group; DSM: Diagnostic and Statistical Manual of Mental Disorders [49]; EX: exposure; FU: Follow-Up; HADS: Hospital Anxiety and Depression Scale;[50] ICG: Inventory of Complicated Grief [51]; [52] (ICG-R); IES: Impact of Events Scale [53]; IG: intervention group; PANAS: Positive Affect Negative Affect Schedule [54]; PCL-C: PTSD-Checklist, Civilian Version [55]; PG-13: Prolonged Grief Scale 13 [18]; PTSD: Posttraumatic Stress Disorder; RRS: Ruminative Response Scale [56]; SF-12: Short Form 12 [57]; STAI: State-Trait Anxiety Inventory [58]; SWLS: Satisfaction With Life Scale [59]; TRIG-D: Texas Revised Inventory of Grief – German Version [60]; UGRS: Utrecht Grief Rumination Scale [61] | | | | | | |
