# Supplementary material for: Effectiveness and Feasibility of Internet-Based Interventions for Grief After Bereavement: Systematic Review and Meta-analysis
Source: JMIR Ment Health. 2021 Dec 8;8(12):e29661. doi: 10.2196/29661 (PMC8701663; doi:10.2196/29661)
Supplement: Multimedia Appendix 4 [file mental_v8i12e29661_app4.docx]

**Meta-Regression Analysis for Grief, PTSS and Depression**

**Grief**

| **Outcome: grief** | **Coeff.** | **SE** | **95% CI** |
| --- | --- | --- | --- |
| Drop out rate (IG) | -.01 | .01 | -.04; .02 |
| Drop out rate (CG) | .02 | .03 | -.05; .09 |
| Therapist feedback *(ref.: no feedback)* | -.05 | .39 | -.81; .72 |
| Number of sessions | .08 | .05 | -.02; .19 |
| Time since loss (months) | .01 | .01 | -.01; .03 |
| Mean age of participants (years) | .00 | .02 | -.03; .03 |

**PTSS**

Meta-regression not possible due to low heterogeneity (I² = 0.00%)

**Depression**

| **Outcome: grief** | **Coeff.** | **SE** | **95% CI** |
| --- | --- | --- | --- |
| Drop out rate (IG) | -.03 | .01 | -.05; .00 |
| Drop out rate (CG) | .04 | .03 | -.03; .11 |
| Therapist feedback *(ref.: no feedback)* | -.58 | .39 | -1.35; .19 |
| Number of sessions | .08 | .05 | -.03; .18 |
| Time since loss (months) | .01 | .01 | -.01; .02 |
| Mean age of participants (years) | -.00 | .02 | -.03; .03 |
