# Supplementary material for: Effectiveness and Feasibility of Internet-Based Interventions for Grief After Bereavement: Systematic Review and Meta-analysis
Source: JMIR Ment Health. 2021 Dec 8;8(12):e29661. doi: 10.2196/29661 (PMC8701663; doi:10.2196/29661)
Supplement: Multimedia Appendix 5 [file mental_v8i12e29661_app5.docx]

**Risk of bias assessment for individual included studies**

| **Brodbeck et al. 2019 [41]** |  |  |
| --- | --- | --- |
| **Risk of bias item** | **Author’s judgement** | **Support for judgement** |
| 1. random sequence generation | low risk | "Randomisation was performed using the True Random Number Generator on Random.org run by the Randomness and Integrity Services Ltd in Dublin" |
| 1. allocation concealment | low risk | allocation list was concealed from participants and investigators |
| 1. blinding of participants and personnel | not applicable |  |
| 1. blinding of outcome assessment | unclear risk | no information provided |
| 1. incomplete outcome data | low risk | analyses using mixed-effects models, estimating parameters for missing values |
| 1. selective reporting | low risk | all pre-defined outcome data reported in "Results"-section |
| 1. other bias | low risk | adherence to invervention assessed, including subgroup analyses |

| **Dominick et al. 2010 [38]** |  |  |
| --- | --- | --- |
| **Risk of bias item** | **Author’s judgement** | **Support for judgement** |
| 1. random sequence generation | low risk | "a total of 68 participants passed ProtoCall’s phone screening, and were randomly assigned to either the treatment group (immediate intervention) or the control group (usual care)" |
| 1. allocation concealment | unclear risk | no further description of allocation and whether it was concealed |
| 1. blinding of participants and personnel | not applicable |  |
| 1. blinding of outcome assessment | unclear risk | no information provided |
| 1. incomplete outcome data | low risk | no drop outs after beginning of the intervention, which might likely be due to the short intervention time span (2 days). |
| 1. selective reporting | low risk | all pre-defined outcome data reported in "Results"-section |
| 1. other bias | low risk |  |

| **Eisma et al. 2015 [35]** |  |  |
| --- | --- | --- |
| **Risk of bias item** | **Author’s judgement** | **Support for judgement** |
| 1. random sequence generation | low risk | Each participant was randomized (simple randomization) in one of our three conditions with a ratio of 3 (exposure) : 3 (behavioral activation) : 2 (waiting list); randomization check to compare treatment- and control groups on all variables |
| 1. allocation concealment | unclear risk | no further description of allocation and whether it was concealed |
| 1. blinding of participants and personnel | not applicable |  |
| 1. blinding of outcome assessment | unclear risk | no information provided |
| 1. incomplete outcome data | low risk | intention-to-treat-analyses, i.e. use of all randomized participants in analyses. Patterns of drop out are reported sufficiently, no differences between completers and drop outs regarding sociodemographic factors, loss-related variables or outcome measures |
| 1. selective reporting | low risk | all pre-defined outcome data reported in "Results"-section |
| 1. other bias | low risk | sufficient definition of drop outs and adherence to intervention |

| **Van der Houwen et al. 2010 [39]** |  |  |
| --- | --- | --- |
| **Risk of bias item** | **Author’s judgement** | **Support for judgement** |
| 1. random sequence generation | unclear risk | first: 1:1 allocation, changed to 2:1 due to high drop out rates in intervention group; however, no information on method of random allocation |
| 1. allocation concealment | unclear risk | no further description of allocation and whether it was concealed |
| 1. blinding of participants and personnel | not applicable |  |
| 1. blinding of outcome assessment | unclear risk | no information provided |
| 1. incomplete outcome data | low risk | intention-to-treat-analyses, i.e. use of all randomized participants in analyses. Patterns of drop out are reported sufficiently, no differences between completers and drop outs regarding sociodemographic factors, loss-related variables or outcome measures |
| 1. selective reporting | high risk | effect sizes for depressive and grief symptoms not reported |
| 1. other bias | low risk | sufficient definition of drop outs and adherence to intervention |

| **Litz et al. 2014 [40]** |  |  |
| --- | --- | --- |
| **Risk of bias item** | **Author’s judgement** | **Support for judgement** |
| 1. random sequence generation | unclear risk | randomization using stratified block design; however, no information on block size and obtainment of the allocation list |
| 1. allocation concealment | unclear risk | no further description of allocation and whether it was concealed |
| 1. blinding of participants and personnel | not applicable |  |
| 1. blinding of outcome assessment | unclear risk | no information provided |
| 1. incomplete outcome data | low risk | intention-to-treat-analyses, i.e. use of all randomized participants in analyses. Patterns of drop out are reported sufficiently |
| 1. selective reporting | low risk | all pre-defined outcome data reported in "Results"-section |
| 1. other bias | low risk | actual time to complete intervention was markedly longer than intended (24.15 weeks; intended time: 6 weeks); however, within-group comparisons investigating the possible confounding effect of time to complete the intervention were conducted, revealing non-significant differences; sufficient definition of drop outs and adherence to intervention |

| **Kersting et al. 2011 [34]** |  |  |
| --- | --- | --- |
| **Risk of bias item** | **Author’s judgement** | **Support for judgement** |
| 1. random sequence generation | low risk | block randomization using an online-number generator |
| 1. allocation concealment | unclear risk | no information provided |
| 1. blinding of participants and personnel | not applicable |  |
| 1. blinding of outcome assessment | unclear risk | no information provided |
| 1. incomplete outcome data | low risk | intention-to-treat-analysis performed on the basis of the ‘last observation carried forward’ principle; additionally, in case of noncompleters, pretreatment scores were carried forward to replace the missing values (TG, n=45; WL, n=33); sufficient information on patterns of and reasons for drop out |
| 1. selective reporting | low risk | all pre-defined outcome data reported in "Results"-section |
| 1. other bias | low risk | no indication of further bias |

| **Kersting et al. 2013 [33]** |  |  |
| --- | --- | --- |
| **Risk of bias item** | **Author’s judgement** | **Support for judgement** |
| 1. random sequence generation | low risk | unstratified randomization using a true randomnumber  service (http://www.random.org). |
| 1. allocation concealment | high risk | open label, conducted by study coordinator |
| 1. blinding of participants and personnel | not applicable |  |
| 1. blinding of outcome assessment | high risk | researchers and therapist were not blinded to the intervention |
| 1. incomplete outcome data | low risk | intention-to-treat-analysis, performed on the basis of the ‘last observation carried forward’ principle; sufficient information on patterns of drop out |
| 1. selective reporting | low risk | all pre-defined outcome data reported in "Results"-section |
| 1. other bias | low risk | no indication of further bias |

| **Wagner et al. 2006 [36]** |  |  |
| --- | --- | --- |
| **Risk of bias item** | **Author’s judgement** | **Support for judgement** |
| 1. random sequence generation | unclear risk | computer-generated randomization, but no further information provided |
| 1. allocation concealment | unclear risk | no information on concealment of allocation |
| 1. blinding of participants and personnel | not applicable |  |
| 1. blinding of outcome assessment | unclear risk | no information provided |
| 1. incomplete outcome data | low risk | intention-to-treat-analysis; sufficient information on patterns of drop out |
| 1. selective reporting | low risk | all pre-defined outcome data reported in "Results"-section |
| 1. other bias | low risk | no indication of further bias |
| **Wagner & Maercker 2007 [37]** |  |  |
| **Risk of bias item** | **Author‘s judgement** | **Support for judgement** |
| 1. random sequence generation | not applicable | FU-data from Wagner et. Al. 2006 |
| 1. allocation concealment | not applicable | FU-data from Wagner et. Al. 2006 |
| 1. blinding of participants and personnel | not applicable |  |
| 1. blinding of outcome assessment | not applicable | FU-data from Wagner et. Al. 2006 |
| 1. incomplete outcome data | low risk | intention-to-treat-analysis; sufficient information on patterns of drop out; dropouts: lower levels of baseline BSI depression, t(24)=3.07, p <.01,  and failure to adapt, t(24)=2.95, p <.01. |
| 1. selective reporting | low risk | all pre-defined outcome data reported in "Results"-section |
| 1. other bias | low risk | no indication of further bias |
